# Distributed Gram-Schmidt orthogonalization with simultaneous elements refinement

**DOI:** 10.1186/s13634-016-0322-6

**Published:** 2016-02-24

**Authors:** Ondrej Slučiak, Hana Straková, Markus Rupp, Wilfried Gansterer

**Affiliations:** 1 TU Wien, Institute of Telecommunications, Gusshausstrasse 25/E389, Vienna, 1040 Austria; 2 University of Vienna, Faculty of Computer Science, Theory and Applications of Algorithms, Währingerstrasse 29, Vienna, 1090 Austria

**Keywords:** Distributed processing, Gram-Schmidt orthogonalization, QR factorization

## Abstract

We present a novel distributed QR factorization algorithm for orthogonalizing a set of vectors in a decentralized wireless sensor network. The algorithm is based on the classical Gram-Schmidt orthogonalization with all projections and inner products reformulated in a recursive manner. In contrast to existing distributed orthogonalization algorithms, all elements of the resulting matrices **Q** and **R** are computed simultaneously and refined iteratively after each transmission. Thus, the algorithm allows a trade-off between run time and accuracy. Moreover, the number of transmitted messages is considerably smaller in comparison to state-of-the-art algorithms. We thoroughly study its numerical properties and performance from various aspects. We also investigate the algorithm’s robustness to link failures and provide a comparison with existing distributed QR factorization algorithms in terms of communication cost and memory requirements.

## Introduction

Orthogonalizing a set of vectors is a well-known problem in linear algebra. Representing the set of vectors by a matrix ${\mathbf {A}}\in \mathbb {R}^{n\times m}$, with *n*≥*m*, several orthogonalization methods are possible. One example is the so-called *reduced QR factorization* (matrix decomposition), **A**=**QR**, with a matrix ${\mathbf {Q}}\in \mathbb {R}^{n\times m}$ having orthonormal columns, and an upper triangular matrix ${\mathbf {R}}\in \mathbb {R}^{m\times m}$ containing the coefficients of the basis transformation [1]. In the signal processing area, QR factorization is used widely in many applications, e. g., when solving linear least squares problems or decorrelation [2–4]. In adaptive filtering, a decorrelation method is typically used as a pre-step for increasing the learning rate of the adaptive algorithm [5], ([6], p. 351), ([7], p. 700).

From an algorithmic point of view, there are many methods for computing QR factorization with different numerical properties. A standard approach is the *Gram-Schmidt orthogonalization algorithm*, which computes a set of orthonormal vectors spanning the same space as the given set of vectors. Other methods include Householder reflections or Givens rotations, which are not considered in this paper.

Optimization of QR factorization algorithms for a specific target hardware has been addressed in the literature several times (e.g., [8, 9]). Parallel algorithms for computing QR factorization, which are applicable for reliable systems with fixed, regular, and globally known topology, have been investigated extensively (e.g., [10–13]).

Besides parallel algorithms, there are two other potential approaches for computation across a distributed network. In the standard—centralized—approach, the data are collected from all nodes and the computation is performed at a fusion center. Another approach is to consider distributed algorithms for fully *decentralized* networks without any fusion center where all nodes have the same functionality and each of them communicates only with its neighbors. Such an approach is typical for sensor-actuator networks or autonomous swarms of robotic networks [14]. Nevertheless, the investigation of *distributed* QR factorization algorithms designed for loosely coupled distributed systems with independently operating distributed memory nodes and with possibly unreliable communication links has only started recently [3, 15, 16]. In the following, we focus on algorithms for such decentralized networks.

### Motivation

The main goal of this paper is to present a novel distributed QR factorization algorithm—DS-CGS—which is based on the classical Gram-Schmidt orthogonalization. The algorithm does not require any fusion center and assumes only local communication between neighboring nodes without any global knowledge about the topology. In contrast to existing distributed approaches, the DS-CGS algorithm computes the approximations of all elements of the new orthonormal basis *simultaneously* and as the algorithm proceeds, the values at *all* nodes are refined iteratively, approximating the exact values of **Q** and **R**. Therefore, it can deliver an estimate of the full matrix result *at any moment* of the computation. As we will show, this approach is, among others, superior to existing methods in terms of the number of transmitted messages in the network.

In Section 2, we briefly recall the concept of a consensus algorithm which we use later in the distributed orthogonalization algorithm. In Section 3, we review the basics of the QR decomposition and existing distributed methods. In Section 4, we describe the proposed distributed Gram-Schmidt orthogonalization algorithm with simultaneous refinements of all elements (DS-CGS). We experimentally compare DS-CGS with other distributed approaches in Section 5 where we also investigate the properties of DS-CGS from many different viewpoints. Section 6 concludes the paper.

### Notation and terminology

In what follows, we use *k* as the node index, $\mathcal {N}_{k}$ denotes the set of neighbors of node *k*, *N* denotes the (known) number of nodes in the network, $\mathcal {E}$ the set of edges (links) of the network, *d*_*k*_ the *k*th node degree ($d_{k}=|\mathcal {N}_{k}|$), $\bar {d}$ the average node degree of the network, and *t* a discrete time (iteration) index.

We will describe the behavior of the distributed algorithm from a network (global) point of view with the corresponding vector/matrix notation. For example, the (column) vector of all ones denoted by **1**, corresponds to all nodes having value 1. In general, we denote the number of rows of a matrix by *n* and the number of columns by *m*. Element-wise division of two vectors is denoted as ${\mathbf {z}} = \frac {{\mathbf {x}}}{{\mathbf {y}}} \equiv \frac {x_{i}}{y_{i}}, \forall i$, element-wise multiplication of two vectors as **z**=**x**∘**y**≡*x*_*i*_*y*_*i*_,∀*i* and of two matrices as **Z**=**X**∘**Y**. The operation ${\mathbf {X}}\circledast {\mathbf {Y}}$ is defined as follows: Having two matrices **X**=(**x**_1_,**x**_2_,…,**x**_*m*_) and **Y**=(**y**_1_,**y**_2_,…,**y**_*m*_), the resulting matrix ${\mathbf {Z}}={\mathbf {X}}\circledast {\mathbf {Y}}$ is a stacked matrix of all matrices **Z**_*i*_ such that ${\mathbf {Z}}_{i}\,=\,({\mathbf {x}}_{1},{\mathbf {x}}_{2},\dots,{\mathbf {x}}_{i})\circ ((\underbrace {1,1,\dots,1}_{i})\otimes {\mathbf {y}}_{i+1})$ (⊗denotes the Kronecker product; *i* = 1,2,…,*m*−1), i.e.,  thus creating a big matrix containing combinations of column vectors: ${\mathbf {Z}}\in \mathbb {R}^{n\times \frac {m^{2}-m}{2}}$. This later corresponds in our algorithm to the off-diagonal elements of the matrix **R**. Also note that all variables with the “hat” symbol, e.g., $\hat {{\mathbf {u}}}(t)$ represent variables that are computed locally at nodes, while variables with the “tilde” symbol, e.g., $\tilde {{\mathbf {u}}}(t)$, are updated based on the information from neighbors.

## Average consensus algorithm

We model a wireless sensor network (WSN) by synchronously working nodes which broadcast their data into their neighborhood within a radius *ρ* (so-called geometric topology). The WSN is considered to be static, connected, and with error-free transmissions (except for Section 5.4 ahead). Although the practicality of synchronicity can be argued [17, 18], we note that it is not an unrealizable assumption [19].

In the following, we briefly review the classical consensus algorithm for computing the *average* of values distributed in a network. Note that the algorithm can be easily adapted to computing a *sum* by multiplying the final average value (arithmetic mean) by the total number of nodes *N*.

The distributed average consensus algorithm computes an estimate of the global *average* of distributed initial data **x**(0) at each node *k* of a WSN. In every iteration *t*, each node updates its estimate using the weighted data received from its neighbors, i.e.,

$$x_{k}(t) = \left[{\mathbf{W}}\right]_{kk}x_{k}(t-1)+\sum_{k^{\prime}\in\mathcal{N}_{k}}\left[{\mathbf{W}}\right]_{kk^{\prime}}x_{k^{\prime}}(t-1) $$

or from a global (network) point of view

(1) $$ {\mathbf{x}}(t) = {\mathbf{W}}{\mathbf{x}}(t-1).  $$

The selection of the *weight matrix***W**, representing the connections in a strongly connected network, crucially influences the convergence of the average consensus algorithm [20–22]. The main condition for the algorithm to converge is that the largest eigenvalue of **W** is equal to 1, i.e., *λ*_max_ = 1, with multiplicity one, and that each row of **W** sums up to 1. It can then be directly shown [20] that the value *x*_*k*_(*t*) at each node converges to a common global value, e.g., average of the initial values.

If not stated otherwise, we use the so-called Metropolis weights [22] for matrix **W**, i.e.,

(2) $$ [\!{\mathbf{W}}]_{ij} = \left\{ \begin{array}{ll} \frac{1}{1+\max\{d_{i},d_{j}\}} & \text{if}\, (i,j)\in \mathcal{E},\\[0.2cm] 1-\sum_{i'\in\mathcal{N}_{i}}[\!{\mathbf{W}}]_{ii'} & \text{if}\, i=j,\\[0.1cm] 0 & \text{otherwise.}\\[-0.08cm] \end{array}\right.  $$

These weights guarantee that the consensus algorithm converges to the average of the initial values.

## QR factorization

As mentioned in Section 1, there exist many algorithms for computing the QR factorization with different properties [1, 23]. In this paper we utilize the QR decomposition based on the *classical* Gram-Schmidt orthogonalization method (in *ℓ*^2^ space).

### Centralized classical Gram-Schmidt orthogonalization

Given matrix ${\mathbf {A}} =~ ({\mathbf {a}}_{1},{\mathbf {a}}_{2},\dots,{\mathbf {a}}_{m}) \in \mathbb {R}^{n\times m}$, *n*≥*m*, classical Gram-Schmidt orthogonalization (CGS) computes a matrix ${\mathbf {Q}}\in \mathbb {R}^{n\times m}$ with orthonormal columns and an upper-triangular matrix ${\mathbf {R}}\in \mathbb {R}^{m\times m}$, such that **A**=**QR**. Denoting

(3) $$ \begin{aligned} &{\mathbf{Q}}=\left({\mathbf{q}}_{1}~~~{\mathbf{q}}_{2}~~~\dots~~~{\mathbf{q}}_{m}\right)\\ &{\mathbf{R}}= \left({}\begin{array}{ccccc} &\langle{\mathbf{q}}_{1}, {\mathbf{a}}_{1}\rangle &\langle{\mathbf{q}}_{1}, {\mathbf{a}}_{2}\rangle &\dots &\langle{\mathbf{q}}_{1}, {\mathbf{a}}_{m}\rangle \\ &0 &\langle{\mathbf{q}}_{2}, {\mathbf{a}}_{2}\rangle &\langle{\mathbf{q}}_{2}, {\mathbf{a}}_{3}\rangle &\dots\\ &\vdots &&\ddots &\dots\\ &0 &\dots &0 &\langle{\mathbf{q}}_{m}, {\mathbf{a}}_{m}\rangle \end{array} \right), \end{aligned}  $$

we have

(4) $$ {\mathbf{q}}_{i} = \frac{{\mathbf{u}}_{i}}{\left\|{\mathbf{u}}_{i}\right\|_{2}}, i=1,2,\dots, m,  $$

and

(5) $$ {\mathbf{u}}_{i} = {\mathbf{a}}_{i}-\sum_{j=1}^{i-1}\frac{\langle{\mathbf{q}}_{j}, {\mathbf{a}}_{i}\rangle}{\langle{\mathbf{q}}_{j}, {\mathbf{q}}_{j}\rangle}{\mathbf{q}}_{j}, \quad i=1,2,\dots, m,  $$

where $\left \|{\mathbf {u}}\right \|_{2} = \sqrt {\sum _{i=1}^{n}{{u_{i}^{2}}}}$ and $\langle {\mathbf {q}}, {\mathbf {a}}\rangle = \sum _{i=1}^{n}q_{i}a_{i}$.

It is known that the algorithm is numerically sensitive depending on the singular values (condition number) of matrix **A** as well as it can produce vectors **q**_*i*_ far from orthogonal when the matrix **A** is close to being rank deficient even in a floating-point precision [23]. Numerical stability can be improved by other methods, e.g., modified Gram-Schmidt method, Householder transformations, or Givens rotations [1, 23].

### Existing distributed methods

Assuming that each node *k* stores its local values ${u_{k}^{2}}$ and *q*_*k*_*a*_*k*_, it is then straightforward to redefine the CGS in a distributed way, suitable for a WSN, by following the definition of the *ℓ*^2^ norm, i.e., $\left \|{\mathbf {u}}\right \|^{2}_{2} ={u_{1}^{2}}+{u_{2}^{2}}+\dots +{u_{n}^{2}}$ (cf. (4)), and inner products, 〈**q**,**a**〉=*q*_1_*a*_1_+*q*_2_*a*_2_+⋯+*q*_*n*_*a*_*n*_ (cf. (5)). The summations can then be computed using any distributed aggregation algorithm, e.g., average consensus [20]^1^ (see Section 2), and asynchronous gossiping algorithms [24], using only communication with the neighbors.

Nevertheless, to our knowledge, all existing distributed algorithms for orthogonalizing a set of vectors are based on the gossip-based *push-sum algorithm* [16, 24]. Specifically in [3], authors used a distributed CGS based on gossiping for solving a distributed least squares problem and in [15], a gossip-based distributed algorithm for *modified* Gram-Schmidt orthogonalization (MGS) was designed and analyzed. The authors also provided a quantitative comparison to existing parallel algorithms for QR factorization. A slight modification of the latter algorithm was introduced in [25], which we use for comparison in this paper. We denote the two Gossip-based distributed Gram-Schmidt orthogonalization algorithms as G-CGS [3] and G-MGS [25], respectively.

Since the classical Gram-Schmidt orthogonalization computes each column of the matrix **Q** from the previous column recursively, i.e., to know vector **q**_2_, we need to compute the norm of **u**_2_ which depends on vector **q**_1_, the existing distributed algorithms always need to wait for convergence of one column before proceeding with the next column. This may be a big disadvantage in WSNs as it requires a lot of transmissions. Also, if the algorithm fails at some moment, e.g., due to transmission errors, the matrices **Q** and **R** are incomplete and unusable for further application.

In contrast, the distributed algorithm proposed in this paper overcomes these disadvantages and computes approximations of all elements of the matrices **Q** and **R** simultaneously. All the norms and inner products are refined iteratively which leads to a significant decrease of transmitted messages, and also the algorithm brings an intermediate approximation of the whole matrices **Q** and **R** at any time instance.

## Distributed classical Gram-Schmidt with simultaneous elements refinement

As mentioned in Section 3.2, the Gram-Schmidt orthogonalization method can be computed in a distributed way using any distributed aggregation algorithm. We refer to CGS based on the average consensus (see Section 2) as AC-CGS. AC-CGS as well as G-CGS [3] and G-MGS [25] have the following substantial drawback.

In all Gram-Schmidt orthogonalization methods, the computation of the norms ∥**u**_*i*_∥ and the inner products 〈**q**_*j*_,**a**_*i*_〉,〈**q**_*j*_,**q**_*j*_〉, occurring in the matrices **Q** and **R**, depends on the norms and inner products computed from the previous columns of the input matrix **A**. Therefore, each node *k* must *wait* until the estimates of the previous norms ∥**u**_*j*_∥ (*j* < *i*) have achieved an acceptable accuracy before processing the next norm ∥**u**_*i*_∥ (a “cascading” approach; see [15]). The same holds also for computing the inner products. We here present a novel approach overcoming this drawback.

Rewriting Eqs. (4) and (5) by a recursion, we obtain

(6) $$\begin{array}{*{20}l} \hat{{\mathbf{q}}}_{i}(t) &= \frac{\hat{{\mathbf{u}}}_{i}(t)}{\sqrt{N\tilde{{\mathbf{u}}}_{i}(t-1)}},& i=1,2,\dots, m, \end{array} $$

(7) $$\begin{array}{*{20}l} \hat{{\mathbf{u}}}_{i}(t) &= {\mathbf{a}}_{i}-{\mathbf{p}}_{i}(t),& i=1,2,\dots, m, \end{array} $$

where $\tilde {{\mathbf {u}}}_{i}(t)$ is the approximation of $1/N\left \|{\mathbf {u}}_{i}\right \|_{2}^{2}{\mathbf {1}}$ at time *t* and

$$ {\mathbf{p}}_{i}(t) = \sum_{j=1}^{i-1}\frac{\tilde{{\mathbf{p}}}^{(2)}_{j+(i-1)(i-2)/2}(t-1)\circ\hat{{\mathbf{q}}}_{j}(t-1)}{\tilde{{\mathbf{q}}}_{j}(t-1)}, $$

with $\tilde {{\mathbf {p}}}^{(2)}_{j+(i-1)(i-2)/2}(t)$ being an approximation of the off-diagonal inner products 1/*N*〈**q**_*j*_,**a**_*i*_〉**1** (∀*j*<*i*) of matrix **R** (cf. (3)) and $\tilde {{\mathbf {q}}}_{j}(t)$ an approximation of 1/*N*〈**q**_*j*_,**q**_*j*_〉**1** at time *t*. Similarly, we define $\tilde {{\mathbf {p}}}^{(1)}_{i}(t)$ to be an approximation of 1/*N*〈**q**_*i*_,**a**_*i*_〉**1**. As we show later, $\tilde {{\mathbf {u}}}_{i}(t)$, $\tilde {{\mathbf {q}}}_{j}(t)$, $\tilde {{\mathbf {p}}}^{(1)}_{i}(t)$, and $\tilde {{\mathbf {p}}}^{(2)}_{j+(i-1)(i-2)/2}(t)$ converge to $1/N\left \|{\mathbf {u}}_{i}\right \|_{2}^{2}{\mathbf {1}}$, 1/*N*〈**q**_*j*_,**q**_*j*_〉**1**, 1/*N*〈**q**_*i*_,**a**_*i*_〉**1**, and 1/*N*〈**q**_*j*_,**a**_*i*_〉**1**, respectively.

Similarly to the state-of-the-art methods (see Section 3.2), we further assume that the matrices ${\mathbf {A}}\in ~\mathbb {R}^{n\times m}$ and ${\mathbf {Q}}\in ~\mathbb {R}^{n\times m}$ are distributed over the network row-wise, meaning that each node stores at least one row of the matrix **A** and corresponding rows of the matrix **Q** and each node stores the whole matrix **R**. In case *n*>*N*, more rows must be stored at the node and each node must sum the data locally before broadcasting to neighbors. Obviously, the data distribution over the network influences the speed of convergence of the algorithm, as can be seen also in the simulations ahead (see Section 5).

Notation **A**_*k*_,**Q**_*k*_(*t*) here represent the rows of the matrices **A** and **Q** at a given node *k* at time *t*. If more rows are stored in one node, **A**_*k*_ and **Q**_*k*_(*t*) are matrices, otherwise they are row vectors. Matrix **R**^(*k*)^(*t*) represents the whole matrix **R** at node *k* at time *t*.

From a *global* (network) point of view, the algorithm is defined in Algorithm 1.



### *Proof of convergence of DS-CGS*.

For the first column, vector *i*=1, $\hat {{\mathbf {u}}}_{1}(t) = {\mathbf {a}}_{1}$, and thus the convergence results of the average consensus, see Section 2, apply, i.e., as *t*→*∞*, the nodes will monotonically reach the common values, i.e., $\tilde {{\mathbf {u}}}_{1}(t)=1/N\|{\mathbf {a}}_{1}\|^{2}_{2}{\mathbf {1}}$ and thus also, $\hat {{\mathbf {q}}}_{1}(t)=\frac {{\mathbf {a}}_{1}}{\|{\mathbf {a}}_{1}\|^{2}_{2}}$, $\tilde {{\mathbf {q}}}_{1}(t)=1/N{\mathbf {1}}$, $\tilde {{\mathbf {p}}}_{1}^{(1)}(t)=1/N\|{\mathbf {a}}_{1}\|^{2}_{2}{\mathbf {1}}$, and $\tilde {{\mathbf {p}}}^{(2)}_{1}(t)=1/N\langle {\mathbf {a}}_{1}, {\mathbf {a}}_{2}\rangle {\mathbf {1}}$.

Furthermore, for all columns *i*>1, all the elements depend only on the first column (*i*=1), e.g., Eq. (7), $\hat {{\mathbf {u}}}_{2}(t)={\mathbf {a}}_{2}-\frac {\tilde {{\mathbf {p}}}^{(2)}_{1}(t-1)\circ \hat {{\mathbf {q}}}_{1}(t-1)}{\tilde {{\mathbf {q}}}_{1}(t-1)}\Big (\vphantom {\frac {\hat {{\mathbf {u}}}_{1}(t)}{\sqrt {N\tilde {{\mathbf {u}}}_{1}(t-1)}}}\Big.$from Eq. (6) $\Big.\hat {{\mathbf {q}}}_{1}(t)\! =~\!\!\frac {\hat {{\mathbf {u}}}_{1}(t)}{\sqrt {N\tilde {{\mathbf {u}}}_{1}(t-1)}}\Big)$. Thus, eventually, $\hat {{\mathbf {u}}}_{2}(t)$ will converge to **u**_2_ (Eq. (5)) and similarly will do all norms and inner products (Eqs. (4) and (5)) of matrix **Q** and **R**.

Intuitively, we can see that as $\tilde {{\mathbf {u}}}_{1}(t)$ converges to its steady state, all other variables converge, with some “delay,” to their steady states as well. We may say that as the first column converges, it “drags” other elements to their steady states. In the worst case, the consequent (following) column starts to converge only when the previous column is fully converged. This behavior differs from the known methods where we have to wait for $\tilde {{\mathbf {u}}}_{1}(t)$ to be converged before computing other terms.

Note that instead of knowing the number of nodes *N* and using it as a normalization constant, we could transmit an additional weight vector $\boldsymbol {\omega }(t)\in \mathbb {R}^{N\times 1}$, i.e., *Ψ*^(0)^(*t*)=***ω***(*t*) and ***Ψ***(*t*)=(*Ψ*^(0)^(*t*),*Ψ*^(1)^(*t*),*Ψ*^(2)^(*t*),*Ψ*^(3)^(*t*),*Ψ*^(4)^(*t*)), such that ***ω***(0)=(1,0,…,0)^⊤^ and Eq. (6) would change only slightly^2^, i.e.,

$$\hat{{\mathbf{q}}}_{i}(t) = \frac{\hat{{\mathbf{u}}}_{i}(t)}{\sqrt{\frac{{\mathbf{1}}}{\boldsymbol{\omega(t)}}\circ\tilde{{\mathbf{u}}}_{i}(t-1)}}. $$

We note that the normalization constant *N* (or ***ω***(*t*), respectively) affects only^3^ the *orthonormality* (columns remain orthogonal but not normalized) of the columns of the matrix **Q**(*t*), and in case only orthogonality is sufficient, as in [26], we can omit this constant. We can, thus, overcome the necessity of knowing the number of the nodes or reduce the number of transmitted data in the network, respectively.

### Relation to dynamic consensus algorithm

The dynamic consensus algorithm is a distributed algorithm which is able to track the average of a time-varying input signal. There exist many variations of the algorithm, e.g., [27–33]. Comparing the proposed DS-CGS algorithm with a dynamic consensus algorithm from [30, 32], we observe an interesting resemblance.

Formulating DS-CGS from a global point of view, i.e.,

$${\mathbf{X}}(t) = {\mathbf{W}}\left[{\mathbf{X}}(t-1) + \triangle{\mathbf{S}}(t)\right], $$

we observe that it is a variant of the dynamic consensus algorithm with an “input signal” **S**(*t*). However, the “input signal” **S**(*t*) in our case is very complicated as it depends on **X**(*t*−1) and **S**(*t*−1) and cannot be considered as an independent signal as it is usually considered in dynamic consensus algorithms. Therefore, it is difficult to analyze the properties of this input signal and convergence conditions of DS-CGS based on the dynamic consensus algorithm. It is also beyond the scope and focus of this paper to analyze this algorithm in general. Nevertheless, some analysis of this type of dynamic consensus algorithm, for a general input signal, together with the bounds on convergence speed, has been conducted in [34].

## Performance of DS-CGS

In our simulations, we consider a connected WSN with *N* = 30 nodes. We explore the behavior of DS-CGS for various topologies: *fully connected* (each node is connected to every other node), *regular* (each node has the same degree *d*), and *geometric* (each (randomly deployed) node is connected to all nodes within some radius *ρ*—a WSN model). If not stated otherwise, the randomly generated input matrix ${\mathbf {A}}\in ~ \mathbb {R}^{300\times 100}$ has uniformly distributed elements from the interval [0,1] and a low condition number *κ*(**A**)=35.7. In Section 5.3.2, we, however, investigate the influence of various input matrices with different condition numbers on the algorithm’s performance.

Also, except for the Sections 5.3.1 and 5.4, for the consensus weight matrix we use the metropolis weights (Eq. (2)).

The confidence intervals were computed from the several instantiations using a bootstrap method [35].

### Orthogonality and factorization error

As performance metrics in the simulations, we use the following:

- *Relative factorization error*—$\frac {\left \|{\mathbf {A}}-{\mathbf {Q}}(t){\mathbf {R}}^{(k)}(t)\right \|_{2}}{\left \|{\mathbf {A}}\right \|_{2}}$ —which measures the accuracy of the QR factorization at node *k*,
- *Orthogonality error*— ∥**I**−**Q**(*t*) ^⊤^**Q**(*t*)∥_2_ —which measures the orthogonality of the matrix **Q**(*t*) (see step 2 of the algorithm).

**Fig. 1:**
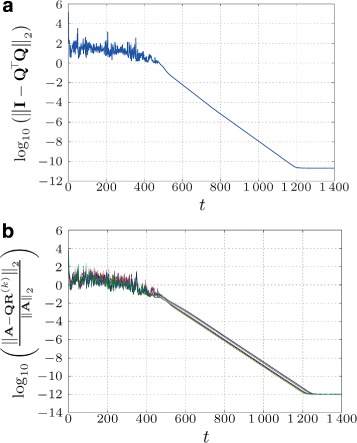
Example of orthogonality (**a**) and factorization error (**b**) for each node k for a geometric topology with *d*=8.533. *N*=30,*k*=1, 2,…,30

Note that both errors are calculated from the network (global) perspective and as depicted, they are not known locally at the nodes, since only **R**^(*k*)^(*t*) is local at each node, whereas **Q**(*t*) is distributed row-wise across the nodes (**Q**_*k*_(*t*)). From now on, we simplify the notation by dropping the index *t* in **Q**(*t*) and **R**^(*k*)^(*t*). The simulation results for a geometric topology with an average node degree $\bar {d}=~8.533$ are depicted in Fig. 1. Since both errors behave almost identically (compare Fig. 1a, b) and since each node *k* can compute a *local factorization error* ∥**A**_*k*_−**Q**_*k*_**R**^(*k*)^∥_2_/∥**A**_*k*_∥_2_ from its local data, we conjecture that such local error evaluation can be used also as a local stopping criterion in practice. Note that this fact was used in [26] for estimating a network size.

Note that the error at the beginning stage in Fig. 1 is caused by the disagreement and not converged norms and inner products across the nodes, i.e., the values of $\tilde {{\mathbf {u}}}(t)$, $\tilde {{\mathbf {Q}}}(t)$, $\tilde {{\mathbf {P}}}^{(1)}(t)$, and $\tilde {{\mathbf {P}}}^{(2)}(t)$. We also observe that the error floor^4^ is highly influenced by the network topology, weights of matrix **W**, and condition number of input matrix **A**. We investigate these properties in Section 5.3.

### Initial data distribution

If *n*>*N*, some nodes store more than one row of **A**. Thus, before doing distributed summation (broadcasting to neighbors), every node has to locally sum the values of its local rows.

**Fig. 2:**
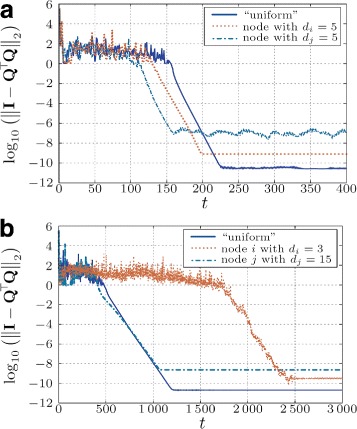
Convergence for networks with different topology and initial data distribution: either all nodes store the same amount of data (“uniform”) or most of the data is stored in one node (with minimum or maximum degree) (**a** - Regular topology with $\overline {d}=5$; **b** - Geometric topology with $\overline {d}=5$). In case of the regular topology (**a**), the nodes *i*,*j* are picked randomly

Simulations show that the convergence behavior of DS-CGS strongly depends on the distribution of the rows across the network (see Fig. 2). We investigate the following cases: (1) each node stores ten rows of **A** (“uniform”); (2) 271 rows are stored in the node with the lowest degree, the other 29 rows in the remaining 29 nodes; and (3) 271 rows are stored in the node with the highest degree, the rest in the remaining 29 nodes.

We observe that not only the initial distribution of the data influences the convergence behavior but also the topology of the underlying network. In the case of a regular topology (Fig. 2a), the influence of the distribution is small and relatively weak in terms of convergence time but stronger in terms of the final accuracy achieved. We recognize that the difference between the nodes comes only from the variance of the values in input matrix **A**. On the other hand, in case of a highly irregular geometric topology (see Fig. 2b), where the node with most neighbors stores most of the data, the algorithm converges much faster than in the case when most of the data are stored in a node with only few neighbors.

**Fig. 3:**
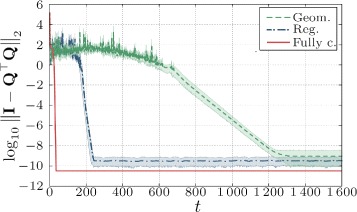
“Uniform” distribution for different topologies. (*Boldface line* is the mean value across six different uniform data distributions. *Shaded areas* are 95 % confidence intervals)

We further observe that in the “uniform” case, the algorithm behaves slightly differently for different distributions of the rows (although still having ten rows in each node). In Fig. 3, we show results for six different placements of the data across the nodes for three different topologies, where we depict the mean value and the corresponding confidence intervals of the simulated orthogonality error. As we can observe, in case of the fully connected topology, the data distribution is of no importance, since all the nodes exchange data in every step with all other nodes. In case of the geometric topology, however, the convergence of the algorithm is influenced by the distribution of data, even if every node contains the same number of rows (ten rows in each node). This can be recognized by bigger confidence intervals of the orthogonality error. Nevertheless, the speed of convergence for all cases is bigger than the case when most data is stored in the “sparsest” node (cf. Fig. 2b). In case of the regular topology, the difference is small only due to numerical accuracy of the mixing parameters.

### Numerical sensitivity

As mentioned in Section 3.1, the classical Gram-Schmidt orthogonalization possesses some undesirable numerical properties [1, 23]. In comparison to *centralized* algorithms, numerical stability of DS-CGS is furthermore influenced by the precision of the mixing weight matrix **W**, the network topology, and properties of input matrix **A**, i.e., its condition number (see Fig. 5 ahead) and the distribution of the numbers in the rows of the matrix (see Figs. 2 and 3). In this section, we provide simulation results showing these dependencies.

#### Weights

**Fig. 4:**
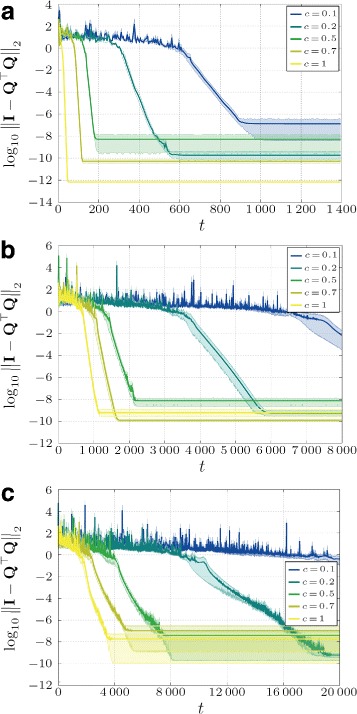
Influence of different constant weights *c* (Eq. (8)) on the algorithm’s accuracy and convergence speed for three different topologies (**a** - Fully connected topology; **b** - Regular topology; **c** - Geometric topology) averaged over ten different input matrices (**a**–**c**). (*Shaded areas* are 95 % confidence intervals)

As mentioned in Section 2, matrix **W** can be selected in many ways. Mainly, the selection of the weights influences the speed of convergence. Unlike previous simulations, where we used the metropolis weights (see Eq. (2)), here we selected constant weights for matrix **W** [20], i.e.,

(8) $$ [{\mathbf{W}}]_{ij} = \left\{ \begin{array}{ll} \frac{c}{N} & \text{if}\, (i,j)\in\mathcal{E},\\[0.1cm] 1-\frac{c}{N}d_{i} & \text{if}\, i=j,\\[0.1cm] 0 & \text{otherwise}, \end{array} \right.  $$

where *c*∈(0,1]. Such weights, in general, lead to slower convergence. However, we can also see in Fig. 4 that the weights influence not only the speed of convergence but also the numerical accuracy of the algorithm (different error floors).

#### Condition numbers

It is well known that the classical Gram-Schmidt orthogonalization is numerically unstable [23]. In cases when input matrix **A** is ill-conditioned (high condition number) or rank-deficient (matrix contains linear dependent columns), the computed vectors **Q** can be far from orthogonal even when computed with high precision.

**Fig. 5:**
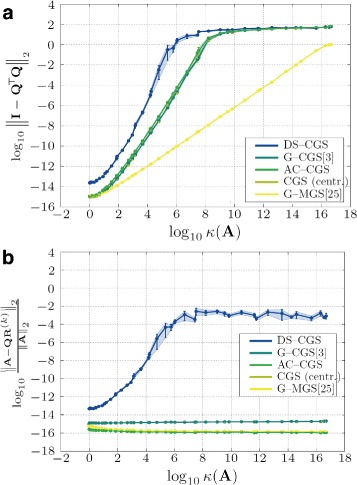
Impact of the condition number *κ*(**A**) of matrix **A** on the orthogonality (**a**) and factorization error (**b**). Averaged over ten matrices for each condition number. Fully connected network. (*Both axes* are in logarithmic scale. *Shaded areas* are 95 % confidence intervals)

In this section, we study the influence of the condition number of input matrix **A** on the accuracy of the orthogonality. The condition number is defined with respect to inversion as the ratio of the largest and smallest singular value. In comparison to classical (centralized) Gram-Schmidt orthogonalization, we observe (Fig. 5a) that the DS-CGS algorithm behaves similarly, although it reaches neither the accuracy of AC-CGS nor of the centralized algorithm (even in the fully connected network). We observe in all of the simulations that the orthogonality error in the first phase can reach very high values (due to divisions by numbers close to zero), which may influence the numerical accuracy in the final phase.

We further observe that the algorithm requires matrix **A** to be very well-conditioned even for the fully connected network. Unlike other methods, the factorization error in case of DS-CGS has the same characteristics as the orthogonality error and is also influenced by the condition number of the input matrix, see Fig. 5b. Although, as we noted in Section 5.1, orthogonality and factorization error of DS-CGS behave almost identically, the dependence of condition number *κ*(**A**) on the factorization error would need a further investigation.

Figure 5 also shows that G-MGS is the most robust method in comparison to the others. This is caused by the usage of the *modified* Gram-Schmidt orthogonalization instead of the classical one.

#### Mixing precision

Another factor influencing the algorithm’s performance is the numerical precision of the mixing weights **W**. Here, we simulate the case of a geometric topology with the Metropolis weights model, where the weights are of given precision—characterized by the number of variable decimal digits (4, 8, 16, 32, “Infinite”).^5^

**Fig. 6:**
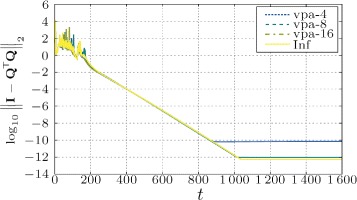
Influence of the numerical precision of the mixing weights on the orthogonality error of DS-CGS. Geometric topology, matrix **A** with low condition number (*κ*(**A**)=1.04)

**Fig. 7:**
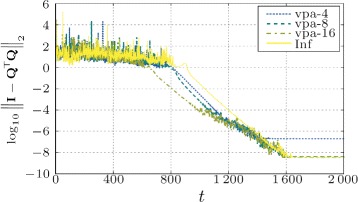
Influence of the numerical precision of the mixing weights on the orthogonality error of DS-CGS. Geometric topology, matrix **A** with higher condition number (*κ*(**A**)=76.33)

**Fig. 8:**
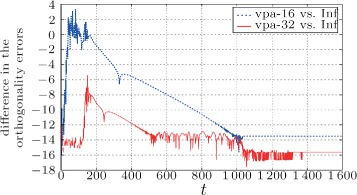
Difference in the orthogonality error $\left (\log _{10}\!\left |\left \|{\mathbf {I}}-{\mathbf {Q}}^{\top }{\mathbf {Q}}\right \|_{2}^{(\text {vpa}-i)}\!-\!\left \|{\mathbf {I}}-{\mathbf {Q}}^{\top }{\mathbf {Q}}\right \|_{2}^{(\text {Inf})}\right |\right)$ for the case of 16 and 32 decimal digits versus “infinite” precision (converted to double)

**Fig. 9:**
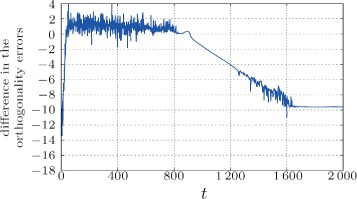
Difference in the orthogonality error $\left (\log _{10}\!\left |\left \|{\mathbf {I}}\,-\,{\mathbf {Q}}^{\top }{\mathbf {Q}}\right \|_{2}^{(\text {vpa}-16)}-\left \|{\mathbf {I}}-{\mathbf {Q}}^{\top }{\mathbf {Q}}\right \|_{2}^{(\text {Inf})}\right |\right)$ for the case of 16 decimal digits versus “infinite” precision (converted to double). Note that in comparison to Fig. 8, the difference between “infinite” and more than 16 digits is below the machine precision (exact same results)

If we compare Fig. 6 with Fig. 7, we find that the numerical precision of the mixing weights have bigger influence in cases when the input matrix is worse conditioned. In Figs. 8 and 9, we can see the difference between orthogonality errors for various precisions. We observe that for the matrix **A** with higher condition number, the higher mixing precision has bigger impact on the result.

As we find in Fig. 6, the error floor moves with the mixing precision. However, we must note that even for the “infinite” mixing precision the orthogonality error stalls at an accuracy (∼10^−12^) lower than the used machine precision—taking into account also the conversion to double precision. From the simulations, we conclude that this is caused by high numerical dynamic range in the first phases of the algorithm as well as by the errors created by the misagreement among the nodes during the transient phase of the algorithm.

### Robustness to link failures

In case of distributed algorithms, it is of big importance that the algorithm is robust against network failures. Typical failures in WSN are message losses or link failures, which occur due to many reasons, e.g., channel fading, congestions, message collisions, moving nodes, or dynamic topology.

We model link failures as a temporary drop-out of a bidirectional connection between two nodes, meaning that no message can be transmitted between the nodes. In every time step, we randomly remove some percentage of links in the network. As a weight model, we picked the constant weights model, Eq. (8), due to its property that every node can compute at each time step the weights *locally* based only on the number of received messages (*d*_*i*_). Thus, no global knowledge is required. However, the nodes must still work synchronously.^6^

**Fig. 10:**
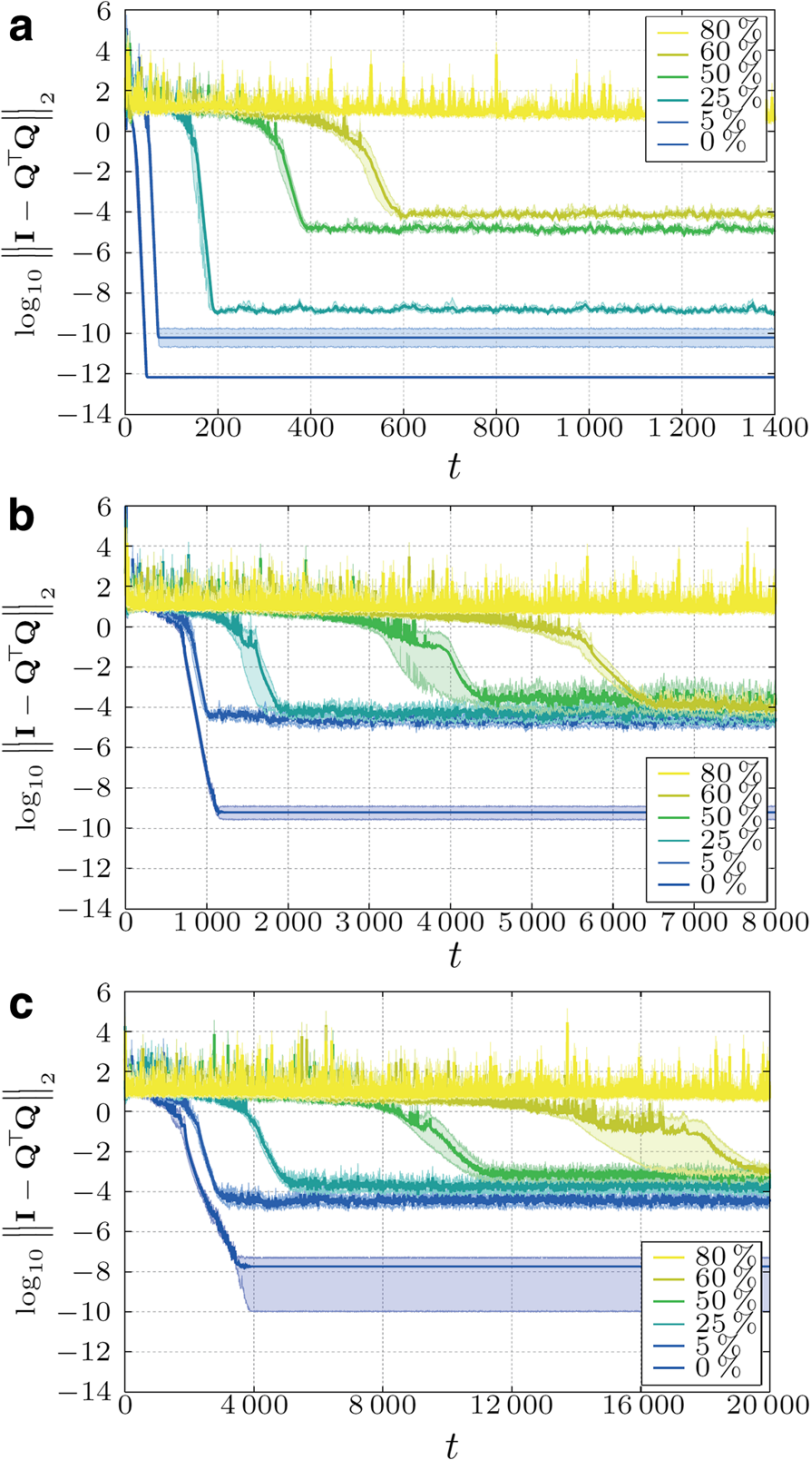
Robustness to link failures for different percentages of failed links at every time step (**a** - Fully connected; **b** - Regular topology; **c** - Geometric topology). Constant weight model with *c*=1, i.e., the fastest option (see Fig. 4). (*Shaded areas* are 95 % confidence intervals)

From Fig. 10, we conclude that the algorithm is very robust and even if we drop in every time step, a big percentage (up to 60 *%*) of the links, the algorithm still achieves some accuracy (at least 10^−2^; Fig. 10c).

It is worth noting that moving nodes and dynamic network topology can be modeled in the same way. We therefore argue that the algorithm is robust also to such scenarios (assuming that synchronicity is guaranteed).

### Performance comparison with existing algorithms

We compare our new DS-CGS algorithm with AC-CGS, G-CGS, and G-MGS introduced in Section 3.2. Although all approaches have iterative aspects, the cost per iteration strongly differs for each algorithm. Thus, instead of providing a comparison in terms of number of iterations to converge, we compare the communication cost needed for achieving a certain accuracy of the result. We investigate the total number of messages sent as well as the total amount of data (real numbers) exchanged.

**Fig. 11:**
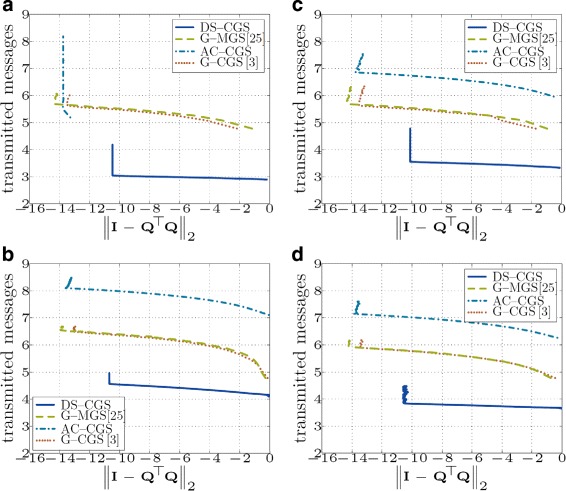
Total number of transmitted messages in the network vs. orthogonality error (*both axes* are in *logarithmic* scale log10) (**a** - Fully connected topology; **b** - Geometric topology with $\overline {d}=8.53$; **c** - Geometric topology with $\overline {d}=24.46$; **d** - Regular topology with $\overline {d}=5$)

**Fig. 12:**
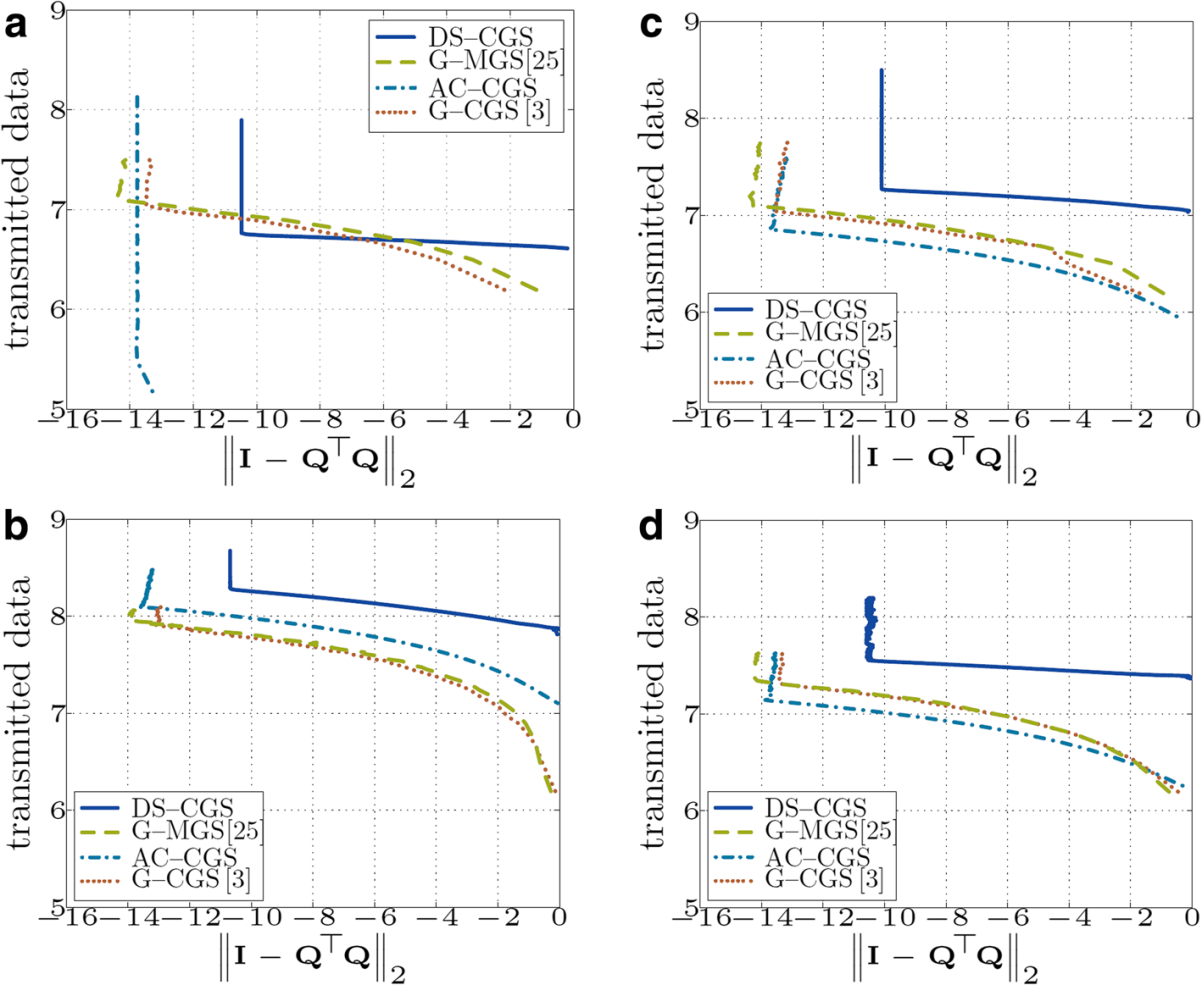
Total number of transmitted real numbers (data) in the network vs. orthogonality error (*both axes* are in *logarithmic* scale log10) (**a** - Fully connected topology; **b** - Geometric topology with $\overline {d}=8.53$; **c** - Geometric topology with $\overline {d}=24.46$; **d** - Regular topology with $\overline {d}=5$)

Simulation results for various topologies are shown in Figs. 11 and 12. The gossip-based approaches exchange, in general, less data (Fig. 12), but since their message size is much smaller than in DS-CGS, the total number of messages sent is higher (Fig. 11).

Because the message size of AC-CGS is even smaller than in the gossip-based approaches, it sends the highest number of messages. Since the energy consumption in a WSN is mostly influenced by the number of transmissions [36, 37], it is better to transmit as few messages as possible (with any payload size); therefore, DS-CGS is the most suitable method for a WSN scenario. However, we notice that in many cases, DS-CGS does not achieve the same final accuracy of the result as the other methods.

Note that in fully connected networks, AC-CGS delivers a highly accurate result from the beginning, because within the first iterations, all nodes exchange the required information with all other nodes.

In Table 1, we summarize the total communication cost and local memory requirements of the algorithms. However, due to different parameters, it is difficult to rank the approaches in a general case. The requirements depend especially on the topology of the underlying network, the number of iterations *I*^(*s*)^ and *I*^(*d*)^ required for convergence in “static” and “dynamic” consensus-based algorithms or the number of rounds *R* needed for convergence of push-sum in the gossip-based approaches. For example, in a *fully connected* network *R*=*O*(log*N*) [24], *I*^(*s*)^=1. Thus, AC-CGS requires *O*(*m*^2^*N*) messages sent as well as data exchanged, whereas gossip-based approaches need *O*(*mN* log*N*) messages and *O*(*m*^2^*N* log*N*) data. Note that G-CGS and G-MGS have theoretically identical communication cost; however, G-MGS is numerically more stable (see Fig. 5) and achieves a higher final accuracy (see Figs. 11 and 12). In case of DS-CGS and a fully connected network, we can interpret DS-CGS in the worst case as *m* consequent static consensus algorithms (one for each column); thus, *I*^(*d*)^=*O*(*m*), and the number of transmitted messages is *O*(*mN*) and data *O*(*m*^3^*N*). Nevertheless, theoretical convergence bounds of DS-CGS (on *I*^(*d*)^) remain an open research question.

**Table 1:** Comparison of various distributed QR factorization algorithms

	Total number of	Total amount of	Local memory
	sent messages	data (real numbers)	requirements
			per node
DS-CGS	*N*·*I* ^(*d*)^	$N\cdot I^{(d)} \cdot \frac {m^{2}+5m}{2}$	*O*(*mn*/*N* + *m* ^2^)
AC-CGS	$N \cdot I^{(s)}\cdot \frac {(m+1)m}{2}$	$N \cdot I^{(s)} \cdot \frac {(m+1)m}{2}$	*O*(*mn*/*N* + *m* ^2^)
G-CGS	*N*·*R*·(2*m*−1)	$N \cdot R \cdot \frac {m^{2}+5m-2}{2}$	*O*(*nm*/*N*)
G-MGS	*N*·*R*·(2*m*−1)	$N \cdot R \cdot \frac {m^{2}+5m-2}{2}$	*O*(*nm*/*N*)

*I* ^(*d*)^ denotes the number of iterations of “dynamic” consensus, *I* ^(*s*)^ the number of iterations of “static” consensus, *R* the number of rounds per push-sum, *N* the number of nodes, *m* the number of columns of the input matrix

## Conclusions

We presented a novel distributed algorithm for computing QR decomposition and provided an analysis of its properties. In contrast to existing methods, which compute the columns of the resulting matrix **Q** consecutively, our method iteratively refines all elements at once. Thus, in any moment, the algorithm can deliver an estimate of both matrices **Q** and **R**. The algorithm dramatically outperforms known distributed orthogonalization algorithms in terms of transmitted messages, which makes it suitable for energy-constrained WSNs. Based on our empirical observation, we argue that the evaluation of the local factorization error at each node might lead to a suitable stopping criterion for the algorithm. We also provided a thorough study of its numerical properties, analyzing the influence of the precision of the mixing weights and condition numbers of the input matrix. We furthermore analyzed the robustness of the algorithm to link failures and showed that the algorithm is capable to reach a certain accuracy even for a high percentage of link failures.

The biggest drawback of the algorithm is the necessity to have synchronously working nodes. This leads to poor robustness when the messages are sent (or lost) asynchronously. As we showed, since the algorithm originates from the classical Gram-Schmidt orthogonalization, also the numerical sensitivity of the algorithm is a big issue and needs to be addressed in the future. The optimization of the weights and design of algorithm in such way that it avoids a big dynamic numerical range, especially in the first phases, is also of interest.

An alternative approach, not considered here, which could be worth of future research, would be to find a distributed algorithm as an optimization problem, e.g., mins.t. **Q**^⊤^**Q**=**I**∥**A**−**QR**∥. In literature, there exist many distributed optimization methods, e.g., [38, 39], which could lead to even superior algorithms, with even faster convergence and smaller error floors.

Last but not least, theoretical bounds of DS-CGS for the convergence time and rate remain an open issue. A first application of the algorithm has already been proposed in [26]. Also, since the proposed algorithm is not restricted to the usage in wireless sensor networks only, a transfer of the proposed algorithm onto so-called network-on-chip platforms [40] could possibly lead to further new interesting and practical applications as well.

## Endnotes

^1^Knowing *n*, $\left \|{\mathbf {u}}\right \|^{2}_{2} =~ n{\lim }_{\textit {t}\to \infty }{\mathbf {W}}^{t}({\mathbf {u}}\circ {\mathbf {u}})=\sum _{i=1}^{n}{u_{i}^{2}}$.

^2^${\lim }_{\textit {t}\to \infty }\boldsymbol {\omega }(t)=1/N{\mathbf {1}}$.

^3^Not considering numerical properties.

^4^Error level at which the algorithm stalls at given computational precision.

^5^The simulations were performed in Matlab R2011b 64-bit using the Symbolic Math Toolbox with variable precision arithmetic. “Infinite” precision denotes weights represented as an exact ratio of two numbers. The depicted result after “infinite” precision multiplication was converted to double precision.

^6^If there is a link, nodes see each other and immediately exchange messages. From a mathematical point of view, this implies that weight matrix **W** will be doubly stochastic [1] in every time step.

## Appendix: local algorithm

For a better clarity, we here reformulate DS-CGS algorithm from the point of view of an individual node *i* (local point of view). Note that input matrix **A** is stored row-wise in the nodes, and for simplicity, we show here the case when the number of rows of matrix ${\mathbf {A}}\in \mathbb {R}^{n\times m}$ is equal to the number of nodes in the network. For a formulation from the network (global) point of view and arbitrary size of matrix **A**, see Section 4.


